# Factors associated with uptake of optimal doses of intermittent preventive treatment for malaria among pregnant women in Uganda: analysis of data from the Uganda Demographic and Health Survey, 2016

**DOI:** 10.1186/s12936-019-2883-y

**Published:** 2019-07-26

**Authors:** Denis Okethwangu, Jimmy Opigo, Stella Atugonza, Catherine T. Kizza, Monica Nabatanzi, Claire Biribawa, Daniel Kyabayinze, Alex R. Ario

**Affiliations:** 1 Uganda Public Health Fellowship Programme, Kampala, Uganda; 2 Uganda National Malaria Control Programme, Ministry of Health, Kampala, Uganda; 3 Malaria Consortium, Kampala, Uganda; 4 Makerere University College of Health Sciences, School of Medicine, Kampala, Uganda

**Keywords:** Malaria in pregnancy, Intermittent preventive treatment, Uganda

## Abstract

**Background:**

The Uganda National Malaria Control Programme recognizes the importance of minimizing the effect of malaria among pregnant women. Accordingly, strategies including intermittent preventive treatment of malaria in pregnancy using sulfadoxine-pyrimethamine (IPTp-SP) have been scaled up. Uptake of IPTp-SP among pregnant women in Uganda, aged 15–49 years who had had a live birth 2 years preceding the 2016 Uganda Demographic and Health Survey (UDHS) was determined and factors associated with the uptake of optimal IPTp-SP doses were identified.

**Methods:**

This was a secondary analysis of the UDHS 2016 dataset. The outcome variable was uptake of IPTp-SP doses among women 15–49 years old who had had a live birth 2 years preceding the survey. Independent variables were residence type, age, marital status, education, wealth status, region of residence, parity, number of antenatal care (ANC) attendance, timing to first ANC visit, and exposure to messages through radio. Logistic regression was used to identify factors associated with the uptake of optimal IPTp-SP doses.

**Results:**

Uptake of three or more doses of IPTp-SP was 18%. The likelihood of taking optimal doses of IPTp-SP was increased among those who had attained a secondary-level education (aOR: 1.5, 95% CI 1.04–2.15), those who attended ANC ≥ 4 times (aOR: 1.34, 95% CI 1.12–1.60), and those exposed to radio messages (aOR: 1.23, 95% CI 1.02–1.48). Among those in the age category > 34 years (aOR: 0.70, 95% CI 0.53–0.92), and those who attended first ANC in the third trimester of pregnancy (aOR: 0.58, 95% CI 0.38–0.87) the odds of uptake were decreased.

**Conclusions:**

Education status, exposure to radio messages about health and frequency of ANC attendance were associated with increased uptake while timing of first ANC attendance and being > 34 years were associated with decreased uptake. The findings suggest a need to strengthen behaviour change communication among women of child-bearing age in order to improve uptake of IPTp-SP during pregnancy.

## Background

Globally, malaria remains a public health threat of concern. In 2016, an estimated 216 million cases of malaria occurred worldwide, a slight rise from 211 million cases in 2015, but a significant drop compared to 237 million cases in 2010. These cases resulted in 445,000 and 446,000 deaths in 2016 and 2015, respectively [1]. Accordingly, over 88% of malaria burden occurs in the African region, with children under 5 years of age and pregnant women bearing the biggest burden [1, 2]. *Plasmodium falciparum* accounts for over 95% of all malaria infections in the continent, the other four parasite species accounting for the remainder [3].

Malaria remains a leading cause of morbidity and mortality in Uganda with over 90% of the population living at risk of developing the disease [4, 5]. Uganda is ranked fourth among the highest malaria-burden countries in the world, with some of the highest transmission rates in the world [6]. Malaria accounts for up to 50% of outpatient visits, 15–20% of admissions and up to 20% of hospital deaths [5]. According to the Uganda Malaria Indicator Survey 2009 in Uganda, malaria parasitaemia was high in most regions of the country, with hyper-endemicity (prevalence of 50–75%) demonstrated in three regions, meso-endemicity (prevalence 10–50%) in six, and hypo-endemicity (prevalence < 10%) in one region [7]. In the next Malaria Indicator Survey conducted in 2015, parasitaemia risk declined in all regions of the country [6, 8]. This decline in parasitaemia has been attributed to interventions, including the increased coverage of insecticide-treated mosquito nets (ITNs) and indoor residual spraying (IRS) [6, 9].

Pregnant women and children are particularly vulnerable because of their reduced immunity [10]. Recognizing the vulnerability of pregnant women to the effects of malaria, the national programme scaled up the availability of ITNs during the first antenatal (ANC) visit, along with sulfadoxine-pyrimethamine (SP) as a preventive therapy during each ANC visit after the first trimester, and appropriate case management through prompt and effective treatment of malaria in pregnant women [4, 11]. The Uganda Ministry of Health guidelines for management of malaria in pregnancy recommend at least three doses of SP for malaria intermittent preventive treatment in pregnancy (IPTp) starting from the second trimester [5, 11]. The policy guidelines on ANC recommends a minimum of four ANC visits during pregnancy [12]. The 2016 Uganda Demographic and Health Survey (UDHS) found that over 90% of women aged 15–49 years who had had a live birth 2 years preceding the survey had received ANC from a skilled personnel, with 60% attending at least four times [13]. The national target for at least three doses of IPTp-SP in Uganda is 80% [14].

Even though SP is recommended to be taken during ANC, the coverage of IPTp-SP does not mirror that of ANC. Uptake of IPTp-SP among pregnant women in Uganda, aged 15–49 years who had had a live birth 2 years preceding the UDHS was determined and factors associated with the uptake of optimal IPTp-SP doses identified.

## Methods

### Study design and data source

This was a cross-sectional study that utilized data from the UDHS 2016. Details of the study design, methods and sampling strategy are described in the UDHS 2016 final report [13]. In summary, the sampling frame for this survey was adopted from the Uganda National Population and Housing Census (NPHC) conducted in 2014. The sample for the survey was selected in two stages. In the first stage, 697 enumeration areas (EAs) were selected from the 2014 NPHC frame: 162 EAs in urban areas and 535 in rural areas. In the second stage, a listing of all households in selected EAs was obtained. Through a systematic random sampling strategy, households were selected for the survey. All women aged 15–49 years who were either permanent residents of the selected households or visitors who stayed in the household the night before the survey were eligible to be interviewed. Data for the survey were collected from 20 June to 16 December, 2016. The data collection tools were translated into eight major languages, namely Ateso, Ngakarimojong, Lugbara, Luganda, Luo, Runyoro-Rutoro, Lusoga, and Runyankole-Rukiga. Of 19,088 eligible women, 18,506 were successfully interviewed, representing a 97% response rate. To limit the effect of recall bias, and in keeping with the Roll Back Malaria indicator on IPTp-SP, the sample was restricted to women who had had a live birth within the 2 years preceding the survey. Therefore, 5901 women were included in this analysis.

### Outcome variable

The outcome variable was uptake of IPTp-SP and was categorized as taking less than three doses or at least three doses as recommended by the World Health Organization (WHO) [11].

### Independent variables

Data on socio-demographic characteristics, including age, marital status, education status, area and region of residence, and wealth index were abstracted. Other variables considered for analysis were parity, exposure to radio messages about health, number of ANC attendances as well as timing of first attendance, and source of anti-malarials during last pregnancy. Age of participants was categorized into 15–24, 25–34 and older than 34 years. Marital status was categorized as never married, married and separated/widowed/divorced. Education status was categorized as no education, primary education, secondary education, and higher education. Participants’ area of residence was either urban or rural. Wealth index scores were derived using the principal component analysis. Households were given scores based on the number and kind of consumer goods they own, including a television set, bicycle or car, and housing characteristics such as a source of drinking water, toilet facilities and flooring material. The computed wealth scores were then divided into five equal categories called quintiles. The lowest quintile comprised the poorest and the highest quintile, the richest. Ugandawas divided into 12 different regions, namely Kampala, Central, Busoga, Bukedi, Teso, Karamoja, Bunyoro/Toro, West Nile, Acholi, Lango, Bugishu, and Ankole/Kigezi. Parity defined as the participants’ number of live births was categorized into 1 child, 2 children and ≥ 3 children. Exposure to radio messages about health was categorized as not at all, less than once a week, and at least once a week.

Data on attendance of ANC were also abstracted, and were assessed using three items, namely number of ANC visits for most recent pregnancy, timing of first ANC visit, and source of anti-malarials during pregnancy. The number of ANC visits was categorized into adequate (≥ 4 visits) and inadequate (< 4 visits) as per WHO recommendation [12]. Timing of first ANC attendance was categorized into first trimester, second trimester and third trimester. Source of anti-malarials was either at ANC, at another facility visit or other sources.

### Statistical analysis

Analyses were conducted using STATA 15 (StataCorp 2017) utilizing the svyset command to match the multistage cluster sampling design method. Distribution of socio-demographic characteristics by IPTp-SP uptake was assessed using the Chi square test. Differences with p-values < 0.05 were considered significant (2-tailed). Both bivariate and multivariable logistic regression analysis were conducted to obtain crude odds ratios (cOR) and adjusted odds ratios (aOR) respectively and their 95% confidence intervals (95% CI). Predictors that were statistically significant (p < 0.05) in the Chi square test were entered in the multivariable logistic regression model. All analyses considered the complex sample design. Sample weighting was used to adjust for the cluster sampling design and sampling probabilities across clusters and strata.

### Ethical considerations

The survey protocol was reviewed and approved by the Inner City Fund International Inc., Fairfax, VA, USA (ICF) Institutional Review Board. To access the survey data, permission from the Demographic and Health Survey Program of the US Agency for International Development was sought and obtained. The data received did not have personal identifiers. In addition, clearance was obtained from the Centers for Disease Control and Prevention, Atlanta, Georgia, USA (CDC), Associate Director for Science, to conduct this study as non-research.

## Results

### Socio-demographic characteristics of respondents

There was a total of 5901 women aged 15–49 years who had had a live birth within the 2 years preceding the survey. Among the respondents, 4629 (78%) took one or more doses of IPTp-SP, 2744 (46%) took two or more doses, and 1049 (18%) took at least three doses. The average age of the respondents was 27 (SE: 0.1) years. The majority of the respondents (79%) lived in urban areas, 4984 (84%) were under 35 years of age, and 4972 (84%) were married. Over 1750 respondents (30%) had attained at least secondary education. There was a similar distribution of participants by wealth quintile, with 1166 (20%) in the richest quintile, 1037 (18%) in the richer quintile, 1119 (19%) in the middle quintile, 1253 (21) in the poorer quintile, and 1326 (22%) in the poorest quintile. The central region had the highest number of participants (1366 [23%]) and Bukedi region had the lowest number (169 [2.8%]).

### Distribution of uptake of IPTp-SP by participant characteristics and risk factors

There was a significant variation among women who got optimal doses and those who did not by age group (*p*-value < 0.01), education status (*p*-value < 0.01) and region of residence (*p*-value < 0.01). Residence (rural or urban), marital status and wealth quintile of respondents were not statistically significant.

Significant variation was also observed among risk factors including parity (*p *= 0.01), number of ANC attendance (*p *< 0.01), source of anti-malarials (*p *= 0.04), timing to antenatal care (*p *< 0.01) and exposure to messages through listening to radio (*p *= 0.01) (Table 1).

**Table 1:** Distribution of socio-demographic characteristics and risk factors among women aged 15–49 years who had a live birth 2 years preceding the survey, by uptake of IPTp doses, UDHS 2016

Characteristic	N = 5901	Participants’ uptake of IPTp		p-value
		< 3 doses n (row %)	≥ 3 doses n (row %)	
Residence				0.37
Urban	1258	1049 (83)	209 (17)	
Rural	4643	3803 (82)	840 (18)	
Age group (years)				< 0.01**
15–24	2511	2020 (80)	491 (20)	
25–34	2473	2037 (82)	436 (18)	
> 34	917	796 (87)	121 (13)	
Marital status				0.661
Never married	371	297 (80)	74 (20)	
Married	4972	4090 (82)	882(18)	
Separated/divorced/widowed	558	465 (83)	93 (17)	
Education				<0.01**
Higher education	432	369 (85)	63 (15)	
Secondary education	1325	1045 (79)	280 (21)	
Primary education	3578	2956 (83)	622 (17)	
No education	566	483 (85)	83 (15)	
Wealth				0.06
Richest	1166	970 (83)	196 (17)	
Richer	1037	840 (81)	197 (19)	
Middle	1119	903 (81)	216 (19)	
Poorer	1253	1017 (81)	236 (19)	
Poorest	1326	1123 (85)	203 (15)	
Region of residence				<0.01**
Kampala	236	200 (85)	36 (15)	
Central	1366	1113 (81)	253 (19)	
Busoga	580	450 (78)	130 (22)	
Bugishu	396	319 (81)	77 (19)	
Acholi	300	249 (83)	51 (17)	
West Nile	412	353 (86)	59 (14)	
Bukedi	168	153 (91)	15 (8.9)	
Lango	302	234 (77)	68 (23)	
Karamoja	282	235 (83)	47 (17)	
Teso	420	338 (80)	82 (20)	
Bunyoro/Toro	800	657 (82)	143 (18)	
Ankole/Kigezi	639	551 (86)	88 (14)	
Parity				0.01**
1 child	1338	1091 (82)	247 (18)	
2 children	1107	878 (79)	229 (21)	
3+ children	3456	2883 (83)	573 (17)	
Number of ANC visits				<0.01**
< 4 times	2228	1898 (85)	330 (15)	
≥ 4 times	3554	2845 (80)	709 (20)	
Unknown	118	109 (92)	9 (7.6)	
Source of anti-malarial				0.04**
Antenatal care	4540	3517 (77)	1023 (23)	
Other facility visit	70	52 (74)	18 (26)	
Other sources	20	13 (65)	7 (35)	
Unknown^a^	1271	1271 (100)	0 (0)	
Timing to ANC				<0.01**
1st trimester	1691	1386 (82)	305 (18)	
2nd trimester	3622	2929 (81)	693 (19)	
3rd trimester	473	428 (90)	45 (10)	
Unknown^a^	115	110 (96)	5 (4.3)	
Listen to radio				0.01**
Not at all	1624	1365 (84)	259 (16)	
< Once a week	889	748 (84)	141 (16)	
≥ Once a week	3388	2740 (81)	648 (19)	

**Significant p-values, < 0.05

^a^Category indicating missing data

### Factors associated with uptake of optimal doses of IPTp-SP

From the bivariate analysis, being older than 34 years (cOR: 0.63, 95% CI 0.49–0.81) and seeking first ANC attendance in the third trimester (cOR: 0.48, 95% CI 0.32–0.70) reduced the chances of uptake of optimal (≥ 3) doses of IPTp-SP among respondents. However, attaining a secondary-level education (cOR: 1.56, 95% CI 1.12–2.18), attending ANC at least 4 times (cOR: 1.43, 95% CI 1.22–1.69), residing in Busoga region (cOR: 1.61, 96% CI 1.01–2.57), and exposure to radio messages at least once a week (cOR: 1.25, 95% CI 1.05–1.49) increased the chances of uptake of optimal doses of IPTp-SP. Belonging to the poorer (cOR: 1.29, 95% CI 1.05–1.57), middle (cOR 1.33, 95% CI 1.06–1.67) and the richer (cOR: 1.30, 95% CI 1.04–1.63) wealth quintiles was also significantly associated with higher odds of uptake of optimal doses of IPTp-SP among the respondents (Table 2).

**Table 2:** Factors associated with uptake of optimal doses of IPTp among women aged 15–49 years who had a live birth within 2 years preceding the survey, UDHS 2016

Characteristic	< 3 doses	≥ 3 doses	cOR (95% CI)	p-value	aOR (95% CI)	p-value
Age group (years)						
15–24	2020	491	Reference		Reference	
25–34	2036	437	0.88 (0.75–1.03)	0.12	0.91 (0.77–1.07)	0.25
> 34	796	121	0.63 (0.49–0.81)*	< 0.01	0.70 (0.53–0.92)**	0.01
Education						
Higher education	369	63	Reference		Reference	
Secondary	1045	280	1.56 (1.12–2.18)*	0.008	1.50 (1.04–2.15)**	0.028
Primary	2956	622	1.23 (0.89–1.70)	0.86	1.27 (0.88–1.81)	0.20
No education	483	83	1.00 (0.68–1.47)	0.99	1.42 (0.93–2.17)	0.11
Marital status						
Never married	297	74	Reference			
Married	4090	882	0.87 (0.62–1.22)	0.42		
Separated	465	93	0.81 (0.53–1.24)	0.34		
Residence						
Urban	1050	208	Reference			
Rural	3803	840	1.11 (0.90–1.38)	0.33		
Wealth						
Poorest	1123	202	Reference		Reference	
Poorer	1017	236	1.29 (1.05–1.57)*	0.015	1.16 (0.93–1.44)	0.19
Middle	903	217	1.33 (1.06–1.67)*	0.013	1.20 (0.93–1.54)	0.17
Richer	840	197	1.30 (1.04–1.63)*	0.022	1.10 (0.85–1.42)	0.47
Richest	970	196	1.12 (0.87–1.45)	0.38	1.02 (0.74–1.40)	0.92
Region of residence						
Kampala	200	36	Reference		Reference	
Central	1113	253	1.27 (0.84–1.92)	0.26	1.23 (0.78–1.94)	0.38
Busoga	450	130	1.61 (1.01–2.57)*	0.046	1.55 (0.93–2.58)	0.092
Bugishu	319	77	1.35 (0.85–2.15)	0.20	1.27 (0.76–2.15)	0.36
Acholi	249	51	1.14 (0.70–1.85)	0.60	1.20 (0.69–2.09)	0.52
West Nile	353	59	0.93 (0.58–1.48)	0.75	0.93 (0.55–1.56)	0.77
Bukedi	153	15	0.54 (0.28–1.06)	0.073	0.56 (0.26–1.21)	0.14
Lango	234	68	1.62 (0.98–2.66)	0.058	1.73 (1.01–2.96)**	0.046
Karamoja	235	47	1.12 (0.68–1.84)	0.65	1.20 (0.69–2.10)	0.52
Teso	338	82	1.36 (0.83–2.21)	0.22	1.35 (0.78–2.34)	0.28
Bunyoro/Toro	657	143	1.21 (0.81–1.83)	0.35	1.19 (0.75–1.89)	0.46
Ankole/Kigezi	551	88	0.89 (0.57–1.41)	0.62	0.83 (0.51–1.38)	0.48
Parity						
1 child	1091	247	Reference			
2 children	878	230	1.15 (0.92–1.45)	0.92		
3+ children	2883	572	0.88 (0.74–1.04)	0.74		
Number of ANC visits						
< 4 times	1898	331	Reference		Reference	
≥ 4 times	2845	709	1.43 (1.22–1.69)*	< 0.01	1.34 (1.12–1.60)**	0.001
Unknown	109	9	0.46 (0.20–1.07)	0.07		
Source of antimalarial						
ANC	3517	1023	Reference			
Other facility visit	52	18	1.19 (0.65–2.17)	0.57		
Other sources	13	7	1.86 (0.69–4.99)	0.22		
Unknown	1271	0	–	–		
Timing of ANC visit						
1st trimester	1386	305	Reference		Reference	
2nd trimester	2929	693	1.07 (0.90–1.28)	0.42	1.13 (0.93–1.36)	0.22
3rd trimester	428	45	0.48 (0.32–0.70)*	< 0.01	0.58 (0.38–0.87)**	0.009
Listen to radio						
Not at all	1365	259	Reference		Reference	
< Once a week	748	142	1.00 (0.76–1.32)	0.99	1.05 (0.79–1.40)	0.73
≥ Once a week	2739	648	1.25 (1.05–1.49)*	0.013	1.23 (1.02–1.48)**	0.028

*Significant variables at the bivariate analysis

**Significant variables at the multivariate logistic regression

In the multivariable analysis, the predictors independently associated with increased uptake of optimal doses of IPTp-SP were secondary-level education (aOR: 1.5, 95% CI 1.04–2.15), residing in the Lango region (aOR: 1.73, 95% CI 1.01–2.96), attending ANC at least four times (aOR: 1.34, 95% CI 1.12–1.60), and exposure to radio messages about health (aOR: 1.23, 95% CI 1.02–1.48). However, belonging to the age category older than 34 years (aOR: 0.70, 95% CI 0.53–0.92), and first attending ANC in the third trimester of pregnancy (aOR: 0.58, 95% CI 0.38–0.87) were associated with decreased uptake of optimal IPTp-SP doses (Table 2).

## Discussion

This study, aimed at identifying factors associated with optimal uptake of IPTp-SP in Uganda, used data from a population-based survey conducted countrywide. It is intended that findings from this study shall inform practice and policy, in order to increase uptake of optimal doses of IPTp-SP. From analyses in this study, the rate of uptake of the optimal dose of IPTp-SP was 18%. Uptake of optimal doses of IPTp-SP among pregnant women in Uganda was associated with education, age group, exposure to radio messages, number of ANC visits, region of residence, and timing to ANC. Pregnant women in the age category older than 34 years and those who attended ANC for the first time in their third trimester were less likely to take optimal doses of IPTp-SP. Pregnant mothers who had attained a secondary-level education, those who are exposed to radio messages at least once a week, and those from Lango region were more likely to take optimal doses of IPTp-SP.

The rate of uptake of optimal doses of IPTp-SP was substantially lower than the 30.2% among pregnant women in Malawi [15], but higher than 11% reported in a study in Tanzania [16]. This rate represents a 39% decline from the rates from an earlier population-based survey conducted in Uganda in 2014/2015 [8]. Findings from this survey indicated that the uptake of two doses was over two and a half times that of at least three doses. Recall that the change of policy from at least two to three doses was rolled out by the WHO in 2013 [11]. While the low uptake of optimal doses of IPTp-SP may be attributed to the recent change in policy, studies need to be conducted to understand the factors for the decline from the immediate previous survey.

The association of ANC attendance with uptake of optimal doses of IPTp-SP has been demonstrated in a number of studies. In Malawi, both timing to the first ANC attendance and frequency of ANC attendance were found to significantly influence uptake of optimal doses of IPTp-SP [15]. In Tanzania, timing to the first ANC attendance increased the chances of optimal IPTp-SP uptake [17]. Similarly, in Kenya, a study conducted to assess factors for uptake of at least two doses of IPTp-SP found that the earlier the onset of ANC visits, the higher the likelihood of completing the required doses [3]. This survey also found that over 95% of pregnant women reported attending ANC at least once from a skilled provider, with 60% attending at least four times; however only 29% had their first ANC attendance in the first trimester [13]. A qualitative study found that some of the reasons for this low IPTp-SP uptake despite the high ANC attendance include lack of training and supervision by healthcare workers, resulting in poor knowledge of IPTp guidelines and uncertainty about the safety and efficacy of SP; inconsistent provision of ANC services leading to services being denies to some women, and drug stock-outs in private healthcare facilities [18, 19].

A study conducted in Uganda to assess factors influencing uptake of at least two doses of IPTp-SP found that pregnant women older than 34 years were less likely to complete required IPTp-SP doses [4]. This finding is in agreement with results from this study that found that respondents 35 years and older were less likely to complete at least three doses. This finding seems rather strange as it is generally expected that older women, especially those who have had earlier pregnancies, would have been exposed to the benefits of IPTp-SP. It contradicts findings from a study conducted in Malawi that showed that lower uptake of IPp-SP was associated with the younger women between 24 and 35 years of age, who may have a poor healthcare-seeking culture [20]. However, a possible explanation may be that the older women having been exposed to sub-optimal quality of care at healthcare facilities, are no longer enthusiastic about utilizing the services. There is need to identify the specific reasons for this finding. Findings from this study show, as it does in Kenya, the effectiveness of exposure to radio messages where listening to health messages at least once a week was associated with increased likelihood of IPTp-SP uptake [3]. Specific communication messages targeting change in behaviour of pregnant women in this age group may likely be effective in changing this trend.

Education as a factor for uptake of IPTp-SP may be related to awareness of malaria prevention programmes. Education beyond the primary level affords respondents a level of exposure that permits them to understand the benefits of IPTp-SP. Indeed one of the factors independently associated with uptake of at least two doses of IPTp-SP in Uganda was awareness of the benefits of IPTp-SP in malaria prevention [4]. An earlier survey also found that 90% of respondents had some knowledge of the benefits of IPTp-SP [8]. Numerous studies have highlighted the association of education and uptake of optimal doses of IPTp-SP [16, 17, 20].

This study had some limitations. The study did not include women who had a stillbirth, or whose children died before birth, considering only those who had a live birth. Including only women with a live birth denied the opportunity to study factors that may have been unique to women who had still births. Also, there was a possible over- or underestimation of information provided by respondents due to recall bias. Recall bias may have been occasioned as self-report was the primary information source. However, limiting the duration to birth 2 years before the survey may have limited the effect of recall bias.

The survey did not address factors related to healthcare service providers or the health system. Factors such as stock-outs of drugs or inadequate access to drugs at healthcare facilities, which have been documented to affect uptake of healthcare services, were left out [20]. Despite the study design that made it impossible to infer causation, this study used a large, unbiased and nationally representative sample from the 2016 demographic and health survey. The large sample size makes the findings of the study generalizable to the entire population.

## Conclusions

The rate of uptake of optimal doses of IPTp-SP among pregnant women between 15 and 49 years of age in Uganda is still low. Taking optimal IPTp-SP doses was associated with a secondary-level education, attending ANC at least four times during the pregnancy, exposure to radio messages about health at least once a week, and residing in the Lango region. However, women older than 34 years of age, and those who attended ANC for the first time in their third trimester were associated with reduced chances of taking optimal IPTp-SP doses. In light of these findings, it is recommended that behaviour change communication to women in the reproductive age groups is intensified, and reasons why despite a high ANC attendance, uptake of IPTp-SP is low need to be explored.

## Data Availability

The data that were used in this study are publicly available with permission from the Demographic and Health Survey Program on http://www.dhsprogram.com/data/dataset.

## Abbreviations

IPTp-SP: intermittent preventive treatment in pregnancy sulfadoxine-pyrimethamine
NMCP: National Malaria Control Program
UDHS: Uganda Demographic and Health Survey
MIS: Malaria Indicator Survey
ANC: antenatal care
aOR: adjusted odds ratio
cOR: crude odds ratio
CI: confidence intervals
SP: sulfadoxine-pyrimethamine
WHO: World Health Organization
ITNs: insecticide treated nets
NPHC: National Population and Housing Census
EAs: enumeration areas
